# Physical and mental growth and development in children with congenital hypothyroidism: a case–control study

**DOI:** 10.1186/s13023-021-02017-7

**Published:** 2021-09-23

**Authors:** Javad Nazari, Kimia Jafari, Maryam Chegini, Akram Maleki, Pari MirShafiei, Ali Alimohammadi, Yasan Kazemzadeh, Reihaneh Mikaeliyan, Saeed Amini

**Affiliations:** 1 Department of Pediatrics, Medical School, Arak University of Medical Sciences, Arak, Iran; 2 Health Deputy, Arak University of Medical Sciences, Arak, Iran; 3 Department of Forensic Medicine, School of Medicine, Arak University of Medical Sciences, Arak, Iran; 4 Department of Health Services Management, Khomein University of Medical Sciences, Khomein, Iran; 5 Department of Health Services Management, Health School, Arak University of Medical Sciences, Arak, Iran

**Keywords:** Physical development, Mental development, Congenital hypothyroidism, Emotional intelligence, Development criteria

## Abstract

**Introduction:**

The clinical complications of congenital hypothyroidism such as brain disorders are very subtle and are not recognizable in infancy period. They are recognizable when it is too late for treatment or prevention. General screening of newborns is effective in diagnosing congenital hypothyroidism and initiating initial treatment. The aim of this study is to compare the physical and mental growth pattern of children with congenital hypothyroidism with healthy ones.

**Methods:**

This case–control study was performed on 34 patients and 68 healthy children who were matched in terms of inclusion and exclusion criteria. Children development screening test (ASQ), children development assessment test (Bayley), preschool Wechsler intelligence scale (WPPSI) and age and steps questionnaire of emotional social development (ASQ-SE) were completed by trained questioners. Data were analyzed using STATA software.

**Results:**

The results indicated that there was no significant difference between the mean of verbal (*P* = 0.77), non-verbal (*P* = 0.81) and general (*P* = 0.66) IQ in permanent and transient patients and healthy individuals. Also, there was no significant difference between the mean of different ranges of ASQ test (including communication, delicate and large movements, problem solving and social) at 12 months and 42 months (P < 0.05). According to Bayley test, there was no significant difference between the cases (permanent and transient) and controls in the cognitive (*P* = 0.42) and expressive (*P* = 0.38) categories. The difference was significant in the perceptual (*P* = 0.011), large (*P* = 0.03) and delicate (*P* = 0.04) movements categories.

**Conclusion:**

This study emphasized on the high effectiveness of neonate hypothyroidism screening program, so that the difference between 3.5 years old children with and without this disease has decreased significantly. Early diagnosis of the patients, while creating beneficial effects for patients and increasing quality of life, cause reduction in the long-term costs of the health system.

## Introduction

Congenital hypothyroidism (CH) is a disease that occurs due to defective production of thyroid hormones from the birth. If the defect occurs in the thyroid glands itself, it is called ''primary hypothyroidism'' and if there is a defect in the release of TSH, it is called ''central hypothyroidism''. Congenital types of hypothyroidism are not inherited and usually occurs due to thyroid dysgenesis. In some cases, hypothyroidism is familial resulting from inborn errors in synthesis of thyroid hormone that usually is due to problems with the synthesis of thyroid hormone with the goiter presentation. Most infants with congenial hypothyroidism are identified in the neonatal screening in the first weeks after birth (first month) before the onset of clinical signs and symptoms of disease [1].

CH is one of the most common predictable causes of mental retardation in children, that early diagnosis and treatment can prevent catastrophic consequences. The clinical complications of congenital hypothyroidism such as brain disorder and injury and nerve growth retardation are very subtle and are not recognizable in infancy and can be recognized when it is too late for treatment or prevention. So, general screening of newborns is effective in diagnosing congenital hypothyroidism and initiating on time initial treatments [2].

Most infants with hypothyroidism are normal at birth and show no signs of the disease. However, follow-up studies in these patients indicate that delays in the diagnosis and treatment, in addition to known neurological complications such as mental retardation, developmental disorders (poor motor coordination, imbalance), eye aberrations, and learning difficulties, may lead to stunted growth pattern and short stature [3, 4].

The incidence rate of CH varies from 1:2000 to 1:4000 in different countries. As an example, this rate is reported to be around 1 patient in 3500 to 5000 in the United States, 1 in 3000 in Europe and 1 in 5700 in Japan [5, 6]. Based on the screening results, the average incidence of the disease in Iran was 2.9 cases per thousand people (1 in 334 live births), 3.8 in the population covered by Arak University of Medical Sciences (1 in 258 live births) [7].

Previous studies have focused mainly on the primary items of children growth such as height and weight and have not addressed developmental issues. In a study by Faizi et al. in 2013, the growth status of children with hypothyroidism that were diagnosed in the congenital hypothyroidism screening program in Isfahan was compared with healthy children in the same age group. In their study, they measured and reported height, weight, and head circumference for case and control groups. The results showed that the range of these indicators in the affected children was significantly different from healthy ones at the beginning of diagnosis, but the disorder in head size decreased to less than 3% of normal head circumference at the age of 3 and height gradually decreased to 3% and 9% of normal height in girls and boys, respectively. However, weight index difference was still more than 3% of normal weight in both sexes. Therefore, it can be concluded that patients with congenital hypothyroidism had growth disorders that was improved during follow-ups [4]. One of the shortcomings of the Faizi et al. study is that they have not studied psychological and developmental indicators of children. In the other study, Khabiri et al. compared the developmental indices of children with congenital hypothyroidism with healthy children using the age and steps questionnaire of ASQ in 2017. According to their results, the mean score of developmental indicators in children with congenital hypothyroidism was significantly lower than the healthy group [8]. In this study, only the ASQ questionnaire was used and other tools and questionnaires to measure physical and mental status were ignored.

There are studies in other countries that have compared developmental status of CH children with healthy ones. Accordingly, SUN et al. in 2012 in Najing, China studied the treatment outcomes of children with congenital hypothyroidism for a 12-year period. They included 163 infected and 163 healthy children in their study. According to the results, height, weight and body mass of children from 6 months to 6 years of age were not significantly different from the control group, although the affected children were at higher risk for overweight and obesity. Affected Children had lower developmental quotient (DQ) than the control group in all aspects of GESell test. Therefore, the researchers have recommended that affected children diagnosed and treated by birth screening have normal growth and neuromotor development [9]. In another study by Van Der Sluijs Veer et al., the quality of life, social development and self-esteem of 69 young people born with congenital hypothyroidism at birth screening in the Netherlands between 1981 and 1982 were assessed using three related questionnaires. The results showed that they have lower quality of life, self- esteem and social development than normal population [10].

The strength point of the present study compared to other studies is that the physical and mental dimensions of children using questionnaires and various tools including ASQ, WPPSI, ASQ- SE, BAELEY tests, as well as children's health files are assessed in the present study. In other words, using these tools, children's verbal, non-verbal and general Intelligence Quotient (IQ), communication scale development, large movements and delicate movements, evolution on a cognitive, linguistic and motor scale, children emotional and social development, indices of head to age ratio, stunting and wasting have been compared between healthy and affected children. Also, the previous studies have not studied separate assessments for transient and permanent hypothyroidism children and have not compared them with healthy ones. They only have investigated the general issue of congenital hypothyroidism [11–13].

Regarding the necessity of conducting this study, firstly, no study has been conducted with the characteristics of the present study previously. The second is that more than 15 years have passed since the implementation of the national program of neonatal hypothyroidism screening in Iran. This necessitate the evaluation of its effectiveness. Therefore, the present study is designed to compare physical and mental development of children with transient and permanent CH with healthy children. This makes possible to accurately describe the current status and therefore provide practical information to health policy makers to redesign this national program.

## Method

### Study population

This is a case–control study that was performed on all three and a half years old children with transient or permanent CH. These children are diagnosed in the national screening program and treatment measures have been performed for them. The number of these children was 34 at the beginning of 2020 in Markazi province. Inclusion criteria included all children aged three and a half years who have been diagnosed and treated as patients in the CH screening program. Exclusion criteria included concurrent infections and diseases such as preeclampsia, intrauterine growth retardation, and genetic problems such as Down syndrome or severe anomalies. The comparison group included healthy three and a half years old children.

### Levothyroxine replacement therapy

On the basis of Neonatal Hypothyroidism Screening Program of Iran, the limit of distinction for primary hypothyroid screening test in neonates is TSH equal to 5 mu/l in the samples taken in the first week of birth and TSH equal to 4 mu/l for the samples taken from day 8 and onwards. Accordingly, for the samples taken in the first week of birth, TSH result lower than 5 mu/l is considered as normal, between 5–9.9 recall for re-screening, and between 10–19.9 recall for confirmation tests including TSH, T3Ru, T4 and/or Free T4, equal or higher than 20 recall for taking intravenous sampling and starting treatment. It is expected that T4 concentration increase to 10 µg/dl during 2 weeks and TSH concentration become normal until 1 month after starting treatment by suitable dose of levothyroxine. The proposed dose for levothyroxine is 10–15 µg/kg/day. However, it is better to start treatment with the dose of 50 µg/day for neonates with very low T4 (equal or lower than 5 µg/dl). Drug interactions and change in levothyroxine dose should be considered [14].

### Sampling method

The case group included all three and a half years old children with transient and permanent CH who were diagnosed through at birth screening tests. This group was included in the study by census. So that, the study sample was equal to the research population (that is 34 patients). Random sampling method was used among healthy three and a half years old children to select the control group. This increases comparability with the case group. For this purpose, twice the number of cases, i.e. 68 people, were selected as the control group by matching the economic and social factors (age, sex, economic and social status of the household, place of residence, etc.). The healthy group were selected using the information contained in the health files of healthcare centers. Based on the indices of age, sex, parents' education, household economic status, the most identical person with the patient in each center was selected and included in the study.

### Administration

Questionnaires were trained on how to measure growth and development of affected and healthy children using the related questionnaires and tools. These questionnaires included second edition of Age and Steps Questionnaire of Social and Emotional development (ASQ-SE-2) developed by Squires et al. (2015) [15]; third edition of child development screening test (ASQ-3) developed by Squires & Bricker (2009) [16]; third edition of developmental assessment test for children (Bayley) developed by Bayley [17]; and third edition of preschool Wechsler intelligence scale (WPPSI-III) developed by Wechsler (2002) [18].

The ASQ screening questionnaires were completed by asking questions from the patient's family, and this way, a score was given to each child for his/her developmental status. The BAELEY developmental test for the children were completed in the specialized center for children growth and development. Finally, the preschool Wechsler intelligence scale (WPPSI) was used. This tool is an individual practice clinical tool that is used to measure the intelligence of children aged 2 years and 6 months to 7 years and three months. This tool is a revised version of the preschool Wechsler IQ scale wppis-r-1989. The Wechsler test includes sub-test scores and total scores that this scores indicate mental performance in the psychological, practical, and verbal ranges. This test has 14 sub-tests including cubes design, information, matrix argument, vocabulary, visual concepts, symbol search, word reasoning, decoding, comprehension, completing images, similarities, word comprehension, joining parts, naming images. The validity and reliability of this test are calculated by Abedi et al. (2015). Based on test–retest reliability, reliability of subsets from 0.44 to 0.94; and using the reliability of the two-half test for subtests, it was in the range of 0.70 to 0.98. The validity of the scale was assessed using the correlation of subtests with each other, and the concurrent validity of the Wechsler revised scale. The correlation coefficients of verbal, practical and total IQs are 0.84, 0.74 and 0.85, respectively, which are very close to the coefficients reported in the main scale guide (0.80, 0.8, and 0.82) [19].

The age and steps questionnaire of emotional social development (ASQ- SE) is an international and standardized questionnaire with high validity and reliability, and its translation has been done by Iran ministry of health to be used in healthcare centers of the country [20]. In this questionnaire, the status of social and emotional intelligence of children is compared with the control group.

Height, weight and head circumference variables are among the periodic care that are measured for all Iranian children and are recorded in children's health files. In this study, trained questioners extracted these indicators from the documents and based on them, BMI, Wasting and stunting indices were calculated.

### Method of analysis

Kolmogorov–Smirnov test was used to assess the normality. This test indicated that P- value is not significant in each one of the studied indicators including verbal, non-verbal and total intelligence, ASQSE, communication ranges, large and delicate movements, problem solving, social, cognitive, perceptual, expressive, dynamic, weight, height, head circumference and BMI. This indicated that all of the indices are normal, so one-way ANOVA test was used to evaluate the mean differences. The difference between two groups was considered significant when the P-value was less than 0.05. Data were analyzed using STATA software version 14.

## Results

About half of the patients lived in Arak city, and the rest lived in the other cities of the province. 91% of patients lived in urban ranges and the rest in villages. More than half of the patients (55.88%) had transient hypothyroidism and the rest had permanent hypothyroidism. 65% of the patients were female and 70% of patients received their treatment before 28 days of age (Table 1).

**Table 1:** Demographic characteristics of the hypothyroidism patients (cases) and healthy individuals (controls 1&2)

Variables	Control 1		Control 2		Cases	
	Number	Percent	Number	Percent	Number	Percent
*N*						
	34	33.3	34	33.3	34	33.3
*Gender*						
Male	27	79.41	9	26.47	22	64.71
Female	7	20.59	25	73.53	12	35.29
*Body mass index (kg/m2)*						
Fat	0	0	1	3.03	1	3.23
Over weight	2	6.25	2	9.09	2	6.45
Normal	30	93.75	30	90.91	28	90.32
*Place of resident*						
Urban	31	91.18	31	91.18	31	91.18
Rural	3	8.82	3	8.82	3	8.82

Mean birth weight (SD) for controls 1, controls 2 and cases was 2770 ± 960, 2850 ± 870 and 3150 ± 1180, respectively. 78%, 72% and 49% of the controls 1, controls 2 and cases had normal delivery, respectively. 7.34% and 4.4% of the cases had history of family thyroid disorders and congenital anomaly, respectively. The control group had no history of family thyroid disorders. TSH level for control groups was between 3.1–5.8 and for the cases was higher than 10. There was no significant correlation between the TSH level and gender (*P* = 0.61). Moreover, there was no significant difference between gender and the prevalence of hypothyroidism (*P* = 0.91). We considered TSH > 10 and free T4 < 1.6 as hypothyroidism.

The results showed that there was no significant difference between the mean of verbal intelligence (*P* = 0.77), non-verbal intelligence (*P* = 0.81) and total intelligence (*P* = 0.66) in the patients (permanent and transient) and healthy individuals (control 1 and 2) (Table 2). Also, no significant difference was observed between the mean of communication category of ASQ at 12 months (*P* = 0.78) and 42 months (*P* = 0.53) in permanent and transient patients and healthy individuals. The results showed that there was no significant difference between the mean of large movements category of ASQ at 12 months (*P* = 0.69) and 42 months (*P* = 0.86) in permanent and transient patients and healthy individuals. According to the results, there is no significant difference between the mean of delicate movements category of ASQ at 12 months (*P* = 0.15) and 42 months (*P* = 0.27) in permanent and transient patients and healthy individuals. The results indicated that there is no significant difference between the mean of problem solving category of ASQ at 12 months (*P* = 0.50) and 42 months (*P* = 0.16) in permanent and transient patients and healthy individuals. According to the results, there is no significant difference between the mean of social range category of ASQ at 12 months (*P* = 0.41) and 42 months at (*P* = 0.29) in permanent and transient patients and healthy individuals (Tables 3, 4).

**Table 2:** Intelligence Coefficient (IQ) scores between controls 1 and controls 2 and cases

Group	SS	DF	MS	F	Prob
*Verbal (IQ)*					
Between groups	39.93	2	17.97	0.06	0.9397
Within groups	27,155.05	97	279.94		
Total	2719.99	99	274.65		
*Nonverbal (IQ)*					
Between *groups*	626.94	2	313.47	1.03	0.36
Within groups	29,598.05	97	305.13		
Total	30,225	99	305.3		
*Total*					
Between groups	142.55	2	71.27	0.31	0.7375
Within groups	22,633.8	97	233.33		
Total	22,776.36	99	230.06		

SS: Sum of Squares; DF: Degree Free; MS: Mean Squares; F: F-Ratio; Prob: Probability

**Table 3:** ASQ categories between controls 1 and controls 2 and cases

Group	SS	df	MS	F	Prob	
*Communication*						
12 Monthly	Between groups	3.92	2	1.96	0.05	0.9511
	Within groups	3364.05	86	39.1		
	Total	3367.97	88	38.27		
42 Monthly	Between groups	10.78	2	5.39	0.5	0.6112
	Within groups	925.29	85	10.88		
	Total	936.07	87	10.75		
*Large movements*						
12 Monthly	Between groups	3926.44	2	1936.22	1.1	0.3383
	Within groups	153,805.57	86	1788.43		
	Total	157,732.02	88	1792.4		
42 Monthly	Between groups	5.72	2	2.86	0.11	0.8986
	Within groups	2230.35	85	26.24		
	Total	2236.07	87	25.7		
*Delicate movements*						
12 Monthly	Between groups	60.75	2	30.37	1.29	0.2797
	Within groups	2020.14	86	23.49		
	Total	2080.89	88	23.64		
42 Monthly	Between groups	138.9	2	69.45	0.73	0.4863
	Within groups	8119.04	85	95.51		
	Total	8257.95	87	49.91		
*Problem solving*						
12 Monthly	Between groups	12.85	2	6.42	0.27	0.7673
	Within groups	2081.52	86	24.2		
	Total	2094.38	88	23.79		
42 Monthly	Between groups	123.01	2	61.5	1.95	0.1490
	Within groups	2684.94	85	31.58		
	Total	2807.95	87	32.27		
*Social*						
12 Monthly	Between groups	123.81	2	61.9	1.46	0.2377
	Within groups	3644.16	86	42.37		
	Total	3767.97	88	42.81		
42 Monthly	Between groups	77.67	2	38.83	0.96	0.3852
	Within groups	3421.19	85	40.24		
	Total	3498.86	87	40.21		

SS: Sum of Squares; DF: Degree Free; MS: Mean Squares; F: F-Ratio; Prob: Probability

**Table 4:** Bayley categories between controls 1 and 2 and cases

Group	SS	df	MS	F	Prob
*Cognitive*					
Between groups	19.65	2	9.82	0.99	0.3763
Within groups	955.51	96	9.95		
Total	975.17	98	9.95		
*Perceptual*					
Between groups	104.8	2	52.4	4.72	0.011
Within groups	1066.48	96	11.1		
Total	1171.29	98	11.95		
*Expression*					
Between groups	11.89	2	5.94	1.34	0.2658
Within groups	425.09	96	4.42		
Total	436.98	98	4.45		
*Delicate movements*					
Between groups	56.98	2	28.49	1.75	0.04
Within groups	1566.42	96	16.31		
Total	1623.41	98	16.56		
*Large movements*					
Between groups	9.71	2	4.58	1.39	0.03
Within groups	335.63	96	3.49		
Total	345.35	98	3.52		

SS: Sum of Squares; DF: Degree Free; MS: Mean Squares; F: F-Ratio; Prob: Probability

According to the results, there was no significant difference between the mean of cognitive category of Bayley test in permanent and transient patients and healthy individuals (*P* = 0.38).

According to the results, there was a significant difference between the mean of perceptual category of Bayley test in permanent and transient patients and healthy individuals (*P* = 0.011). Bonferroni and Scheffe tests were used to determine the location of difference in this category. Both two tests indicated difference between cases, control 1 and control 2 groups, as well as between permanent patients and healthy individuals.

According to the results, there was no significant difference between the mean of expression category (*P* = 0.26) in Bayley test in permanent and transient patients and healthy individuals.

According to the results, there was a significant difference between the mean of delicate (*P* = 0.04) and large movements (*P* = 0.03) categories of Bayley test in permanent and transient and healthy patients. Bonferroni and Scheffe tests were used to determine the difference place in this categories. Both tests showed difference between permanent patients and healthy individuals.

The 3rd, 15th, 50th, 85th, and 97th percentiles for height, weight, and head circumference of both sexes were determined and compared with the control group and WHO values (Table 5). The percentiles of weight, height, and head circumference of studied patients were not significantly different from control group and WHO values (*P* > 0.01). Accordingly, there was no significant difference between the mean of weight to age ratio in permanent and transient patients and healthy individuals (*P* = 0.50), between the mean of height to age ratio in permanent and transient patients and healthy individuals (*P* = 0.35), between the mean of weight to height ratio in permanent and transient patients and healthy individuals (*P* = 0.40), and between the mean of BMI in permanent and transient patients and healthy individuals (*P* = 0.12). However, there was a significant difference between the mean of head circumference to age in permanent and transient patients and healthy individuals (*P* = 0.04). Bonferroni and Scheffe tests to determine the location of the difference in this category indicated that both tests show no difference between permanent patients and healthy people.

**Table 5:** Percentile of head circumference (cm), weight (gr) and height (cm) for age for permanent and transient patients, and healthy and WHO values

	3rd		15th		50th		85th		97th	
	F	M	F	M	F	M	F	M	F	M
*Head*										
Permanent p	46.3	47.3	47.3	48.0	48.8	49.5	50.3	51.3	51.3	52.6
Transient P	46.3	47.1	47.6	48.5	49.4	49.5	50.5	51.4	51.5	52.6
Healthy ones	46.4	47.6	47.7	48.6	49.4	49.9	50.7	51.3	51.4	52.7
WHO values	46.3	47.2	47.5	48.4	49.0	49.9	50.4	51.4	51.6	52.6
*Weight*										
Permanent p	11.4	11.6	12.9	13.8	15.2	14.1	16.2	17.1	18.8	19.0
Transient P	11.8	12.1	13.4	14.2	14.9	15.7	17.6	17.7	18.5	19.6
Healthy ones	11.6	11.6	13.5	14.7	15.3	16.5	17.1	17.5	19.1	19.6
WHO values	11.8	12.2	13.1	13.5	15.0	15.3	17.3	17.5	19.5	19.4
*Height*										
Permanent p	89.4	91.6	94.1	94.9	98.7	99.3	103.0	104.2	105.6	104.7
Transient P	88.7	92.6	95.4	92.7	97.5	96.8	103.5	105.8	104.1	105.6
Healthy ones	91.3	92.4	95.0	95.3	99.4	99.8	103.7	104.5	106.9	107.5
WHO values	91.4	92.4	94.8	95.7	99.0	99.9	103.3	104.0	106.7	107.3

F: Female; M; Male, P: Patients, WHO: World Health Organization

WHO data available at: https://www.who.int/tools/child-growth-standards/standards

## Discussion

In the absence of congenital hypothyroidism treatment, patients will have linguistic, functional and full-scale intelligence lower than healthy individuals. One of the outstanding results of this study is emphasis on effectiveness of pediatric hypothyroidism screening program. So that, comparing 3.5 years old affected and healthy children indicated that there is no significant difference between verbal, nonverbal and general IQ as well as categories of communication, large and delicate movements, problem solving and social ASQ at 12 and 42 months, and also categories of cognitive, expressive and delicate of Bayley test in transient and permanent hypothyroidism and healthy children. These indicates that therapeutic measures for children with transient and permanent hypothyroidism have been done well and the integration of neonatal hypothyroidism screening program in Iran health system in 2005 has been successful. In other words, like the vaccination program for children, which has been carried out in all cities and villages of the country for decades ago, this program is also carried out throughout the country. This program has paved the way for the implementation of other congenital metabolic diseases such as phenylketonuria and fauvism [21, 22].

This study showed that early therapies, in addition to improving IQ and cognitive skills, and individual-social development are effective also in improving motor functions, motor perceptual skills and visual perception, and executive functions such as memory, planning, speech and language. Early detection of affected people, while creating beneficial effects for patients and increasing their quality of life, reduces the long-term costs of the healthcare system.

In spite of highly effective on time cognitive and curative treatments performed for CH patients, but the study indicated that there is a significant difference between the affected and healthy children regarding the categories of perceptual, large and delicate movements of Bayley and also head circumference. According to the Rahmani et al. study, despite timely treatment of CH cases, but children with permanent hypothyroidism have a higher IQ deficit than transient ones. So that Children with PCH had 4 times less intelligent than children with TCH [23]. These cases indicate the necessity of performing reforms on national program of CH screening on the backgrounds of on time treatment and also supervision on its processes.

Previous case–control studies that have compared people with congenital hypothyroidism and healthy children only have focused on the CH risk factors. In the other words, these studies have examined what underlying factors are present in affected children that are not present in healthy ones and thus attributed the cause of the disease to those factors. While in the present study, this issue was investigated that to what extent CH children after several years of diagnosis and treatment are as healthy as normal children in terms of various physical and intelligence variables [24, 25].

The important point about congenital hypothyroidism screening is to use a same and standard method all over the country and world in order to achieve good results. At the moment, different methods such as TSH measurement at baseline following T4 measurement backup, T4 measurement at baseline following TSH measurement and simultaneous TSH and T4 measurement are used for CH screening. This indicates that there is no same protocol globally for this work [26]. Inside Iran, also, depending on the child place of birth and parents’ knowledge, attitude and practice, the screening process may be performed incompletely, non-standard or in inappropriate time in different private and public healthcare centers. Also, the order of child birth, the child sex, distance to healthcare centers, the mother age and pregnancy care, health system coverage and follow-up measures are highly effective on the children CH screening [11]. This emphasizes more than ever the importance of information exchange and cooperation between the various public and private sectors in the provision of services. In addition, different countries use different cut points to screening for hypothyroidism. In Greece, for example, infants with TSH more than 20 are considered as suspicious cases [27]. In Egypt, however, the amount more than 15 are considered for further evaluation [28]. In the Iranian screening program, which started from 2005, TSH value greater than 5 is considered as cases needing more follow-up measures [21]. Another important point about preventing CH is intervention on the changeable factors. A study in Iran showed that consanguineous marriages, low birth weight, type of delivery, infant hospitalization (for various reasons) have significant effects on the CH development. These indicate that screening program alone in the public healthcare system is not enough to preventing CH development, but also it is necessary to integrate all private and first, second, and third levels of healthcare system in order to provide a comprehensive view of the issue. Primary Health Care (PHC) system of Iran has indicated its effectiveness, efficiency and hence productivity in different conditions and crises [29]. Improving social capital and KAP of parents, and supporting low socioeconomic groups are effective in the successful prevention measures [30]. Fortunately, prevention and treatment of CH similar to other PHC services does not need expensive and complicated equipment and is presentable affordable within the current facilities [31, 32].

## Conclusion

This study showed that the program of neonatal screening for early detection and treatment of CH cases has achieved great successes. However, as the study indicated, three and a half years old CH children in comparison with their healthy counterparts have some limited problems in developmental categories. In order to reduce the complications of congenital hypothyroidism in the affected children, it is necessary to implement the screening, treatment and follow up measures uniformly and coherently across the country on the basis of WHO and national guidelines. Identifying the weaknesses and threats of the program and providing information on its shortfalls can inform policymaking and channel resource allocations.

## Data Availability

The datasets analyzed during the current study are available from the corresponding author on reasonable request.
