# Correction to: Physical and mental growth and development in children with congenital hypothyroidism: a case–control study

**DOI:** 10.1186/s13023-021-02131-6

**Published:** 2021-12-07

**Authors:** Javad Nazari, Kimia Jafari, Maryam Chegini, Akram Maleki, Pari MirShafei, Ali Alimohammadi, Yasan Kazemzadeh, Reihaneh Mikaeliyan, Saeed Amini

**Affiliations:** 1 Department of Pediatrics, Medical School, Arak University of Medical Sciences, Arak, Iran; 2 Health Deputy, Arak University of Medical Sciences, Arak, Iran; 3 Department of Forensic Medicine, School of Medicine, Arak University of Medical Sciences, Arak, Iran; 4 Department of Health, Khomein University of Medical Sciences, Khomein, Iran; 5 Department of Health Services Management, Health School, Arak University of Medical Sciences, Arak, Iran

## Correction to: Nazari et al. Orphanet J Rare Dis (2021) 16:393 10.1186/s13023-021-02017-7

Following the publication of the original article [1], it was brought to our attention that two errors were introduced to the authors’ affiliations during the typesetting and implementation of the authors’ corrections:

1. The last and corresponding author Saeed Amini is affiliated with institutions 4 and 5, as shown in author list of this Correction, and not with institutions 3 and 5, as initially shown in the original article.
2. Furthermore, institution 4 is the Department of Health, Khomein University of Medical Sciences, Khomein, Iran, as shown in the “Author details” section of this Correction, and not the Department of Health Services Management, Khomein University of Medical Sciences, Khomein, Iran, as initially shown in the original article.

The original article has already been corrected as above.

## References

[1] Nazari J (2021). Physical and mental growth and development in children with congenital hypothyroidism: a case–control study. Orphanet J Rare Dis.

